# Factors Influencing Turning and Its Relationship with Falls in Individuals with Parkinson’s Disease

**DOI:** 10.1371/journal.pone.0093572

**Published:** 2014-04-03

**Authors:** Fang-Yu Cheng, Yea-Ru Yang, Chung-Jen Wang, Yih-Ru Wu, Shih-Jung Cheng, Han-Cheng Wang, Ray-Yau Wang

**Affiliations:** 1 Department of Physical Therapy and Assistive Technology, National Yang-Ming University, Taipei, Taiwan; 2 Department of Neurology, Cheng Hsin General Hospital, Taipei, Taiwan; 3 Department of Neuromuscular Disorder, Chang Guan Memorial Hospital, Linkou, Taiwan; 4 Department of Neurology, Mackay Memorial Hospital, Taipei, Taiwan; 5 Department of Neurology, Shin Kong Wu Ho-Su Memorial Hospital, Taipei, Taiwan; University Of São Paulo, Brazil

## Abstract

**Background:**

Falls are a major problem for people with Parkinson’s disease (PD). Many studies indicate that more than 50% of people with PD have difficulty in turning that may lead to falls during daily activities. The aims of this study were to identify the relationship between turning performance and falls, and to determine the factors that influence turning performance.

**Methods:**

This study examined 45 patients with idiopathic PD (Hoehn and Yahr stage 1–3) using a battery of tests, including 180° turn time, balance, and muscle strength. The levels of disease severity and freezing of gait were also measured. The number of falls in the past 6 months was recorded.

**Results:**

Sixteen out of forty-five participants experienced falls in the past 6 months. A receiver operating characteristic curve showed that turn time was highly related to falls [more affected side: sensitivity = 0.81, specificity = 0.79, area under the curve (AUC) = 0.83; less affected side: sensitivity = 0.88, specificity = 0.76, AUC = 0.83]. The most important factor influencing turn time was balance ability (both sides: p = 0.000) according to the regression model. Correlations between turn time and dynamic balance were further established with reaction time, movement velocity, endpoint excursion, and maximal excursion of the LOS (limits of stability) test.

**Conclusion:**

The time needed to complete a 180° turn during the SQT (step/quick turn) test is a good index to differentiate fallers from non-fallers in persons with PD. Turn time is most influenced by balance. Furthermore, balance control, especially in an anterior or sideways direction, is important for turning performance.

## Introduction

Turning is an essential part of goal-directed locomotion that people engage in daily. However, turning difficulty is a common problem in people with Parkinson’s disease (PD). A previous study noted that more than 50% of people with PD have difficulty turning that can lead to falls [1]. Compared with regular walking, falls while turning are eight times more likely to result in hip fracture [2]. Therefore, it is important to understand turning performance in people with PD.

According to a literature review, individuals with PD usually move more slowly [3], have a narrower base of support [4], and take more steps to turn than those without PD [4]. Mak and Pang noted that the time up and go (TUG) test could be used to distinguish fallers from non-fallers [5]; a longer TUG time (≥16 seconds) is independently associated with an increased risk of falls in patients with PD. However, the TUG test assesses several other mobility functions in addition to turning activity. Therefore, the influence of turning performance on fall incidence is not known. The first goal of this study was to investigate whether turning performance can differentiate fallers from non-fallers in individuals with PD.

Although turning difficulties are common in people with PD, factors influencing turning performance have not yet been documented. Freezing of gait, postural instability, and decreased muscle strength of the lower extremities have been identified to influence gait performance in patients with PD [6]. However, whether these factors influence turning performance has not yet been established. The second goal of this study was to identify the most important factors influencing turning performance. If the most important factors can be identified, details about these factors can be further analyzed to provide a better understanding and possible future training strategies for improving turning performance.

## Methods

### Participants

Participants were recruited from a medical center in Taiwan. Eligible participants were diagnosed with idiopathic PD by a neurologist. All participants met the following inclusion criteria: (a) Hoehn and Yahr stages I through III, (b) ability to walk independently, (c) stable medication usage, and (d) a score of greater than or equal to 24 on the mini-mental state examination. The exclusion criteria were as follows: (a) unstable medical conditions and (b) histories of other diseases known to interfere with participation in the study. Finally, 53 out of 85 individuals were identified as potential participants for this study. Among these individuals, 45 participants participated in the study.

Information about age, gender, the more affected side, disease duration, and the numbers of falls in the past 6 months were obtained from patient interviews and medical charts. In this study, the more affected side was determined by the history of the limb that was initially affected [7], and falls were defined as events in which the individual unintentionally came to rest on the ground or the lower level that were not the results of other neurological diseases or dizziness [8].

### Ethics Statement

The study protocol was approved by the Institutional Review Board of Taipei City Hospital and explained to all participants prior to their participation. All of the participants had capability to consent (MMSE≥24) and gave written informed consent.

### Study Protocol

The assessments included turning performance, dynamic balance performance, muscle strength, disease severity, and the state of gait freezing. All assessments were conducted with patients in the “on” status. The participants took their medications one hour before the assessment, and the whole assessment procedures were finished within 2 hours to ensure participants were in “on” status.

### Assessments

1. **Turning performance.** Turning performance was assessed according to the protocol of the step/quick turn (SQT) test of the Balance Master system (NeuroCom International, Inc, USA). This system provides two long forceplates to quantify the vertical forces exerted by the subject’s feet. Subjects were asked to take two steps forward on cue, quickly turn 180°, and walk back to the starting position. The measured turn time is the time to execute the 180° turning and the turn sway is the velocity during the 180° turning. The turning sway quantifies the postural stability of the subjects during the turning and is expressed as the average center of gravity (COG) sway velocity in degrees/second [9], [10]. All subjects performed three SQT tests on the more- and less-affected sides.
2. **Balance performance.** Balance performance was assessed by the Tinetti assessment tool for balance and the limit of stability (LOS) test of the Balance Master system. The Tinetti assessment tool is an easily implemented test that measures balance ability and gait. The balance section consists of a 10-item rating for balance performance on tasks. Scores range from 0 to 1 or 0 to 2 for each item, and higher scores indicate better performance [11], [12].

The LOS test provides objective measurements to quantify dynamic balance. To assess the LOS, subjects stood on the forceplate with arms hanging naturally beside the trunk and were instructed to shift his/her center of gravity (COG) to reach the maximal distance in the target direction as quickly and accurately as possible without moving their feet. The COG position on the forceplate was visually expressed on the computer screen as a small humanoid. By shifting their weight in the target direction, participants would move the humanoid representation of their current COG position toward that target.

The target directions included anterior, anterior more-affected side, more-affected side, posterior more-affected side, posterior, posterior less-affected side, less-affected side, and anterior less-affected side. Participants were instructed to return to the starting position and wait for a cue for the next direction. Reaction time (RT), movement velocity (MVL), endpoint excursion (EPE), maximum excursion (MXE), and directional control (DCL) were collected during the LOS test. RT is the amount of time between the command to move and the patient’s first movement that is related to the cognitive function or motor disease [13]. MVL is defined as the average speed of COG movement in degrees per second that is related to the postural preparation ability or bradykinesia [14], [15]. EPE is the distance of the first movement toward the designated target. MXE is defined as the furthest distance traveled by the COG during the trial. Both of these two variables are related to the ability of moving COG toward the target directions that are important for gait performance [16]. DCL is defined as a comparison of the amount of movement in the intended direction to the amount of extraneous movement that is related to the capability of controlling movement.

1. **Muscle strength.** Muscle strength was evaluated using a handheld dynamometer (PowerTrack II; JTech Medical, USA). All tests done were ‘make’ tests in which the dynamometer was held stationary by the examiner while the subjects exerted a maximum force against it. Three trials were obtained with a 1-min rest between trials. The muscle groups measured included trunk flexors, trunk extensors, hip flexors, hip extensors, hip abductors, hip adductors, knee flexors, and knee extensors. The procedures were according to the Bohann’s study [17].

The intra-rater reliability of the muscles in our sample was determined to be good with intraclass correlation coefficients ranging from 0.93 to 0.98. Muscle strength was normalized to body weight, and the average muscle strength of the left and right sides was used for each muscle group. The muscle strength of the hip flexors and knee flexors and of the hip extensors and knee extensors were added to indicate the strength of the flexors and extensors of the lower extremities, respectively.

### Disease Severity and the State of Freezing

Disease severity was determined by a neurologist using the Unified Parkinson’s Disease Rating Scale III (UPDRS III, Motor Section). The UPDRS III is a 14-item rating of Parkinsonian motor signs and symptoms, each item scores from 0 to 4, with higher scores indicating a worse symptom. The state of freezing was assessed by the freezing of gait questionnaire (FOGQ). Freezing of gait is defined as the inability to generate a valid step when experiencing an abrupt episode, such as gait initiation or suddenly turning. The FOGQ has good test-retest reliability [18], with higher scores indicating a serious state of freezing.

### Statistical Analysis

Statistical Package for the Social Sciences 16.0 software was used for data analysis. The relationship between falls and turn time was examined by receiver operating characteristic (ROC) curves, and the area under the curve (AUC) was used to evaluate the effectiveness of turn time in classifying fallers and non-fallers. Youden’s index (sensitivity + specificity –1) was used to determine the optimal cut-off point [19]. The values ranged from 0 to 1, with values closer to 1 indicating better performance in terms of both sensitivity and specificity. Pearson’s correlation coefficient was used to examine correlations between factors and turning time. A linear regression model (stepwise strategy) was used to determine the contribution of disease severity, freezing of gait, balance performance, and muscle strength to turn performance. Statistical significance was set at p<0.05.

## Results

Forty-five subjects (male: 29; female: 16) participated in the study. Table 1 presents basic information and disease characteristics of all participants. Their mean age was 64.3 years (range, 49–84) with a mean PD duration of 6.1 years (range, 1–15) and a Hoehn and Yahr level of 1.8 (range, 1–3). Sixteen patients had fall experiences in the past 6 months (range, 1–13 falls), and 29 patients did not. The average UPDRS III score was 16.5 (ranging 7–33), and the mean FOGQ score was 9.7 (range, 0–24). The mean Tinetti balance score was 13.7 (range, 2–16).

**Table 1:** Demographic and disease characteristics.

Variable	All subjects (N = 45) mean±SD (range)
Age (years)	64.3±9.8 (49–84)
Gender (male/female)	29/16
Disease duration (years)	6.1±4.7 (1–15)
Hoehn-Yahr stage (stage)	1.8±0.8 (1–3)
UPDRS-III (score)	16.5±7.5 (7–33)
FOGQ (score)	9.7±7.1 (0–24)
Tinetti balance (score)	13.7±3.0 (7–16)
Turn time-more affected side (seconds)	3.5±2.2 (0.56–10.40)
Turn time-less affected side (seconds)	3.1±2.9 (0.38–10.85)

Abbreviations: UPDRS, Unified Parkinson’s Disease Rating Scale; FOGQ, freezing of gait questionnaire; SD, standard deviation.

### Discriminating Fallers from Non-fallers

In the SQT test, participants with PD needed more time (more affected side: 3.5±2.2 seconds; less-affected side: 3.1±2.9 seconds) to complete a turning task than an age-matched control subject (1.1 seconds for both sides obtained from NeuroCom databank). According to ROC analysis, the turn time of the SQT test had good discernment to classify fallers. A cut-off point of 2.75 seconds for the more-affected side resulted in a sensitivity of 0.81, a specificity of 0.79, and an AUC of 0.83. A cut-off point of 2.29 seconds for the less-affected side resulted in a sensitivity of 0.88, a specificity of 0.76, and an AUC of 0.83.

### Factors Determining Turn Time

According to the above results, we noted that turn time toward either side could discriminate fallers from non-fallers. Therefore, the next step was to identify the most important factor(s) in determining turn time. The correlations were first established and factors correlating significantly with turn time were further analyzed using a linear regression model. Table 2 shows the correlation results. The UPDRS III and FOGQ scores were positively correlated with turn time (more-affected side: r = 0.65 and r = 0.50, respectively, p<0.05; less-affected side: r = 0.68 and r = 0.55, respectively, p<0.05). The Tinetti balance score was negatively correlated with turn time (more-affected side: r = −0.73, p<0.05; less-affected side: r = −0.85, p<0.05). The muscle strength of the lower extremity extensors and abductors were also negatively correlated with turn time (more-affected side: r = −0.40, p<0.05, r = −0.27, p>0.05; less-affected side: r = −0.47, and r = −0.32, respectively, p<0.05). According to the regression model, the Tinetti balance score was the most important factor in determining turn time (more affected side: F = 45.95, p<0.001, statistics power = 0.99; less affected side: F = 103.08, p<0.001, statistics power = 0.99; Table 3). However, there are many components affecting the balance ability, therefore we further analyzed the balance ability and turn time for possible treatment strategies to improve turning ability and reduce risk of falls. The LOS test, measured in this study, provides objective measurements for dynamic balance. Thus the correlations between the LOS test, the quantified balance performance, and the turn time were examined.

**Table 2:** Correlation coefficients between turn time and factors.

	Turn time, more-affected side	p value	Turn time, less-affected side	p value
UPDRS-III	0.65	<0.001*	0.68	<0.001*
FOGQ	0.50	<0.001*	0.55	<0.001*
Tinetti balance	−0.73	<0.001*	−0.85	<0.001*
Trunk flexor	−0.35	0.019*	−0.45	0.002*
Trunk extensor	−0.16	0.305	−0.20	0.187
LE flexor	−0.18	0.248	−0.20	0.195
LE extensor	−0.40	0.015*	−0.47	0.001*
LE abductor	−0.27	0.072	−0.32	0.034*
LE adductor	−0.13	0.385	−0.29	0.226

Abbreviations: UPDRS, Unified Parkinson’s Disease Rating Scale; FOGQ, freezing of gait questionnaire; LE, lower extremity; *p<0.05.

**Table 3:** Summary of linear regression.

Variable	R	R^2^	β	t	p
More-affected side					
Tinetti balance	0.723	0.522	−0.723	−6.779	<0.001*
Less-affected side					
Tinetti balance	0.843	0.711	−0.843	−10.153	<0.001*

Variables eliminated from the regression model (more-affected side): Unified Parkinson’s Disease Rating Scale (UPDRS III), freezing of gait questionnaire (FOGQ), trunk flexors, and lower extremity (LE) extensor (p>0.05).

Variables eliminated from the regression model (less-affected side): UPDRS III, FOGQ, trunk flexors, LE extensor, and LE abductor; (*p>0.05).

### Dynamic Balance Performance and Turn Time

Table 4 shows the correlation coefficients between turn time and variables of the LOS test. RTs toward the anterior, anterior more-affected, more-affected, and anterior less-affected sides were positively correlated with turn time (more-affected side: r = 0.30–0.52, p<0.05; less-affected side: r = 42–50, p<0.05). MVLs of the anterior, anterior more-affected, more-affected, posterior, posterior less-affected, and anterior less-affected sides were negatively correlated with turn time [more-affected side: r = −0.35−(−0.41), p<0.05; less-affected side: r = −0.38−(−0.45), p<0.05]. The EPEs of the anterior, anterior more-affected, more-affected, posterior more-affected, less-affected, and anterior less-affected sides were negatively correlated with turn time [more-affected side: r = −0.32− (−0.65), p<0.05; less-affected side: r = −0.36−(−0.68), p<0.05]. The MXEs of seven directions were negatively correlated with turn time except for the posterior side [more-affected side: r = −0.33−(−0.65), p<0.05; less-affected side: r = −0.39−(−0.71), p<0.05].

**Table 4:** Correlation coefficients between turn time and limits of stability.

Turn time		
LOS-RT	More affected side	Less affected side
Anterior side	0.49 (p<0.01)	0.42 (p = 0.01)
Anterior, more-affected side	0.52 (p<0.01)	0.47 (p<0.01)
More-affected side	0.30 (p = 0.05)	0.25 (p = 0.11)
Anterior, less-affected side	0.41 (p = 0.01)	0.50 (p<0.01)
**LOS-MVL**		
Anterior side	−0.35 (p = 0.02)	−0.40 (p = 0.01)
Anterior, more-affected side	−0.37 (p<0.01)	−0.43 (p<0.01)
More-affected side	−0.38 (p = 0.01)	−0.41 (p = 0.01)
Posterior side	−0.36 (p = 0.02)	−0.38 (p = 0.01)
Posterior, less-affected side	−0.36 (p = 0.02)	−0.41 (p = 0.01)
Anterior, less-affected side	−0.41 (p = 0.01)	−0.45 (p<0.01)
**LOS-EPE**		
Anterior side	−0.37 (p = 0.01)	−0.36 (p = 0.02)
Anterior, more-affected side	−0.49 (p<0.01)	−0.52 (p<0.01)
More-affected side	−0.65 (p<0.01)	−0.68 (p<0.01)
Posterior, more-affected side	−0.32 (p = 0.03)	−0.36 (p = 0.02)
Less-affected side	−0.37 (p = 0.01)	−0.46 (p<0.01)
Anterior, less-affected side	−0.41 (p = 0.01)	−0.46 (p<0.01)
**LOS-MXE**		
Anterior side	−0.50 (p<0.01)	−0.53 (p<0.01)
Anterior, more-affected side	−0.46 (p<0.01)	−0.54 (p<0.01)
More-affected side	−0.65 (p<0.01)	−0.71 (p<0.01)
Posterior, more-affected side	−0.33 (p = 0.03)	−0.39 (p = 0.01)
Posterior side	−0.36 (p = 0.02)	−0.43 (p<0.01)
Posterior, less-affected side	−0.38 (p = 0.01)	−0.44 (p<0.01)
Less-affected side	−0.52 (p<0.01)	−0.59 (p<0.01)
Anterior, less-affected side	−0.59 (p<0.01)	−0.64 (p<0.01)

Abbreviations: LOS, limit of stability; RT, reaction time; MVL, movement velocity; EPE, endpoint excursion; MXE, maximal excursion; p<0.05.

## Discussion

Results from this study indicate that turn time from either the more- or less-affected side assessed by the SQT test is a good measure for fall discrimination. This means that if turn times are greater than 2.74 seconds from the more-affected side or 2.29 seconds from the less-affected side, patients have a high risk of fall. Furthermore, balance performance is the most influential factor for turning performance compared with disease severity, freezing of gait, and muscle strength.

In our study, turning time could discriminate fallers from non-fallers. Compared to non-fallers, fallers needed more time to complete turning in the SQT test (see below). The SQT test can also evaluate sway during turning. Sway was defined as the average sway (in degrees) of the COG during the 180° turn. The mean value of sway in fallers was significantly greater than in non-fallers (more-affected side, for example, 65.6° *vs.* 52.4°, p<0.05), indicating that the faller was less stable during turning. One needs good control of their COG over their base of support to turn safely, especially at high velocity. We noted that fallers not only turn slowly but also in an unstable manner. It has been suggested that more than 3 seconds to complete a 180° turn indicates turning difficulty in elderly subjects [20]. In our study, fallers needed more than 3 seconds (mean, more-affected side = 5.1 seconds; less-affected side = 4.9 seconds) to complete a 180° turn, indicating difficulties in turning, but not non-fallers (mean, more-affected side = 2.2 seconds; less-affected side = 2.1 seconds). The TUG test has also been reported to distinguish fallers from non-fallers with a sensitivity of 0.69 and a specificity of 0.62 in the PD population [12]. Performance of a 180° turn in our study can better discriminate fallers from non-fallers than the TUG test. The TUG test involves many tasks, including getting up from a chair, walking 3 m, turning 180°, and sitting down; however, the SQT test used in our study only assessed turning 180°. We therefore suggest that turning ability is a more important influence on the occurrence of falls than other abilities tested in the TUG test.

Regarding the turn time needed to complete a 180° turn, we noted that the balance ability tested by the Tinetti balance scale was a more important influencing factor than disease severity, freezing of gait, or lower extremity muscle strength. To further document the relationship between dynamic balance control and turning performance, the LOS test was administered. Our results showed a moderate-to-good relationship between RT and turn time. Prolonged RT correlates with cognitive impairment and/or motor disease. Evidence from another study showed that RT was delayed in individuals with PD [13]. In this study, we also noted that RT was longer in those with PD than in age-matched controls, for example, toward the anterior direction in the LOS test (1.13 *vs.* 0.91 seconds). The longer RT in individuals with PD may indicate a deficit in motor programming for motor execution [21]. However, cognitive impairment and bradykinesia noted in PD cannot be ruled out. MVL in six directions correlated with turn time. MVL in those with PD toward the anterior (2.5 degrees/second), and more-affected (3.5 degrees/second), for example, were slower than in age-matched controls (4.3 degrees/second and 4.9 degrees/second, respectively, from the Balance Master databank). When participants executed the LOS test, they used postural strategies comprising postural preparatory and executive phases [14], [15]. Postural preparation enables subjects to implement stability limits with short RTs and fast speed [15]. Reduced MVL might be due to poor performance during the postural preparatory phase, which has been thought to maintain the COG over the stance limb and preprogram the response for predictable disturbances of postural stability [22]. It is noted that patients with PD have impairments in motor planning and in switching from one motor program to another, which may result in insufficient times for the postural preparatory to executive turning phases [23], [24]. Without good postural preparation, patients may trade off speed for balance during movement. The bradykinesia characterized by PD may impair the executive phase of the LOS test as well as turning performance.

EPE and MXE also had moderate-to-good correlations with turn time. When turning, people must move their COG forward to the targeted side, and the lower extremities must then take an asymmetric step to redirect the cyclical motion [25]. A decreased forward COG EPE led to decreased stride length, gait velocity [16], and turning speed in our study. Due to a SQT assessment of turning toward both sides, COG control toward the anterior and lateral sides at the beginning of turning was needed to complete the entire task. Therefore, excursions toward the anterior and lateral sides were important to turning performance.

Regarding DCL, there was no significant correlation with turn time. Interestingly, when compared with age-matched data, our PD participants performed with more accuracy, similar to the findings of Jessop et al [26]. A possible reason for better performance in DCL is that PD patients may focus on the “as accurate as possible” command, whereas normal subjects focus on the “as quickly as possible” command during the LOS test. Therefore, performance of DCL in the LOS test may not be a good index for dynamic balance control in persons with PD.

### Clinical Implication and Study Limitations

According to our results, the time needed to complete a 180° turn in the SQT test is a good index to differentiate fallers from non-fallers in persons with PD. If turn time from the more-affected side is greater than 2.75 seconds and greater than 2.29 seconds from the less-affected side, there is an increased risk of fall. Turn time is most influenced by balance ability. Furthermore, balance control, especially toward the anterior and sideway direction, are important for turning performance. We suggest that training programs that focus on improving balance control and turning ability will decrease risk of falls in persons with PD. The major limitation of this study is the relatively small sample size. Future studies should have a larger sample size to confirm our results. Moreover, in addition to the factors identified in the present study, there are other factors such as disease stage, disease subtype, comorbidities, cognitive function and the number of years of formal education, which may also affect the balance ability and turning performance. Treatment effects of the balance training to improve turning performance and decrease fall risks are warranted and encouraged for future studies.

## Conclusion

This study suggests that turn time from both sides assessed by the SQT test is a good tool to discriminate fallers from non-fallers, and balance ability is the most influential factor for turning performance compared with disease severity, freezing of gait, and muscle strength in individuals with PD.
